# Alpha-Glucosidase of *Manduca sexta* Is an Entry Factor for *Daphnis nerii* Cypovirus-23

**DOI:** 10.3390/v18030293

**Published:** 2026-02-28

**Authors:** Jian Yang, Wendong Kuang, Zhihao Duan, Zhigao Zhan, Jinchang Wang, Junhui Chen, Feiying Yang, Limei Guan, Jianghuai Li, Huiyun Song, Liang Jin

**Affiliations:** 1 Institute of Biomanufacturing, Jiangxi Academy of Sciences, Nanchang 330029, China; jemappelleyangjian@zju.edu.cn (J.Y.); kuangwendong@163.com (W.K.); zgzhan_iom@163.com (Z.Z.); wangjinchang75@163.com (J.W.); allenchen0426@gmail.com (J.C.); jxaasyfy@163.com (F.Y.); glmnh@126.com (L.G.); jeremy_leakey@sina.com (J.L.); huiyunsong@126.com (H.S.); 2 College of Life Sciences, Nanchang Normal University, Nanchang 330029, China; zhduan1@163.com

**Keywords:** *Daphnis nerii* cypovirus-23, VP3, alpha-glucosidase, virus entry

## Abstract

*Daphnis nerii* can severely damage pine forests worldwide. *Daphnis nerii* cypovirus-23 (DnCPV-23) is an important viral pathogen for controlling *D. nerii*. However, the mechanism underlying DnCPV-23 cell entry has not been elucidated. In this study, we determined that VP3 mediates the binding of DnCPV-23 to host brush border membrane vesicles. Far-Western blotting and mass spectrometry results revealed that a *Manduca sexta* alpha-glucosidase (MsAGL) can interact with VP3. The interaction between MsAGL and VP3 was verified by co-immunoprecipitation and glutathione S-transferase pull-down assays. Notably, MsAGL influenced DnCPV-23 entry into host cells, including attachment and the subsequent internalization of the virus. Furthermore, MsAGL inhibited DnCPV-23 infections of *M. sexta* cells and *D. nerii* larvae. In summary, we confirmed that VP3 of DnCPV-23 mediates cell entry, while also identifying MsAGL as an entry factor for DnCPV-23. The study findings provide useful insights relevant for further elucidating the cell entry mechanisms of cypoviruses.

## 1. Introduction

Cypoviruses (CPVs), which belong to the genus *Cypovirus* in the family *Reoviridae*, typically have an icosahedral capsid layer and a genome comprising 10–11 double-stranded RNA (dsRNA) segments [1]. CPVs infect the midgut of host insect larvae and severely damage the digestive system, resulting in appetite loss, slow movement, and bradygenesis, ultimately leading to death [2]. *Daphnis nerii* cypovirus-23 (DnCPV-23) was first isolated from naturally infected *Daphnis nerii* larvae [3]. The DnCPV-23 genome consists of 10 dsRNA segments (*S1* to *S10*), of which *S1* encodes the major capsid protein; *S2* encodes an RNA-dependent RNA polymerase (RdRP); *S10* encodes a polyhedrin (PH); *S3*, *S5*, *S7*, and *S8* encode different structural proteins; *S4* encodes a minor capsid protein similar to the VP3 protein of Bombyx mori cypovirus (BmCPV)-1; and *S9* encodes a non-structural protein with an unknown function [3]. Given that the RdRP and PH amino acid sequences of DnCPV-23 are phylogenetically located on separate branches, DnCPV-23 is identified as a new CPV that differs from other CPVs, including BmCPV and Dendrolimus punctatus cypovirus (DpCPV) [4]. The receptors and viral proteins mediating the cell entry of DnCPV-23 have not been studied.

For many animal viruses, cellular entry commences with attachment to specific host cell surface receptors [5,6]. This critical first step is then followed by viral internalization, which occurs via the pathways of endocytosis or membrane fusion. Within the endosome, acidic pH or proteolytic enzymes induce conformational changes in viral proteins, leading to membrane fusion or capsid disassembly [7,8]. Finally, the viral genome is released into the cytoplasm or transported into the nucleus, marking the completion of entry and the onset of replication [5,6,9]. An entry factor is a host-derived protein or molecule that is exploited by a virus to facilitate its internalization into the host cell [10,11]. Entry factors may function as primary receptors, co-receptors, attachment factors, or endocytic modulators that directly participate in viral attachment and internalization, virus uncoating or conformational changes [11]. In the family *Reoviridae*, the cell entry mechanism of mammalian *Orthoreovirus* (MRV), rotavirus (RV), and grass carp reovirus (GCRV) have been thoroughly analyzed. MRV attaches to the host cell surface via the σ1 protein, initially establishing low-affinity binding to α-sialic acid (SA) through the σ1 tail [6,9]. Subsequently, a conformational change to σ1 leads to a high-affinity binding to junctional adhesion molecule-A [12], followed by integrin-mediated viral entry into host cells [13]. Nogo-66 receptor 1 (NgR1) mediates the entry of MRV into non-susceptible neuron cells [14]. A recent study indicated that murine neuropilin 1 serves as a receptor for MRV by interacting with outer capsid protein s3 and capsid turret protein λ2 of MRV [15]. Other studies identified paired immunoglobulin-like receptor B and NgR1 as potential receptors for MRV [16,17]. The initial attachment of RV to host cells involves an interaction between the subunit VP8 of turret protein VP4 and cellular SA [18]. The subunit VP5 of turret protein VP4 then binds to integrin α2β1, ultimately mediating the cell entry of RV [19]. A heat shock cognate protein (hsc70) [20], occludin, and ZO-1 [21] have also been identified as receptors that allow RV to enter host cells. A laminin receptor interacts with viral outer capsid protein VP5 to mediate the cell entry of GCRV [22]. Considering it binds to viral outer capsid proteins VP5, VP56, and VP35, cell surface heparan sulfate is an attachment-related receptor for GCRV [23]. Heat shock protein 70 (HSP70) has a key function associated with GCRV entry into host cells [24]. Bluetongue virus (BTV) is an important member of the family *Reoviridae*. SA functions as the host receptor and interacts with the outermost spike-like viral protein VP2 during BTV entry into cells [25]. An earlier study involving *Culicoides variipennis* showed that a 23 kDa membrane protein, which is another receptor candidate, can interact with viral spike protein VP4 during BTV cell entry [26].

Although CPVs also belong to the family *Reoviridae*, there has been limited research on the mechanism mediating its entry into host cells. Integrin beta, receptor for activated protein kinase C (RACK1), and claudin-2 may form a receptor complex during the cell entry of BmCPV [27,28]. Cell membrane ganglioside GM2 and cholesterol also contribute to BmCPV cell entry [29]. Alkaline phosphatase is a candidate receptor for the MTase domain of the viral turret protein B-spike during the cell entry of DnCPV-23 [30]. Another study determined that HSP70, glutamate dehydrogenase, and an angiotensin-converting enzyme of *B. mori* might be components of the receptor complex mediating the cell entry of DnCPV-23 through interactions with a viral spike protein [31]. CPVs attach to the host peritrophic membrane to initiate infections, with host columnar cells the primary targets [32]. The brush border membrane, distributed on the surface of columnar epithelial cells, contains receptors for various viruses and toxins. Brush border membrane vesicles (BBMVs) are microvesicles generated by isolating and purifying this membrane, which then spontaneously form sealed vesicles under experimental conditions [32,33]. They serve as a key model system for studying membrane protein function and transmembrane transport mechanisms, making them a suitable material for identifying receptors specific to DnCPV-23.

In this study, we determined that the VP3 protein encoded by *S4* in the DnCPV genome is a spike protein that contributes to viral cell entry. We further demonstrated that an alpha-glucosidase (MsAGL) interacts with VP3. Notably, DnCPV-23 cell entry, including attachment and internalization were significantly inhibited by a small interfering RNA (siRNA)-based decrease in MsAGL. By contrast, overexpressing MsAGL enhanced viral attachment and internalization. In vitro neutralization bioassays showed that MsAGL can inhibit the ability of DnCPV-23 to infect host cells and partially neutralize the DnCPV-23 infection of *D. nerii*. On the basis of the study findings, MsAGL mediates the entry of DnCPV-23 into host cells.

## 2. Materials and Methods

### 2.1. Viruses, Insects, Cell Lines, and Antibodies

DnCPV-23, which was originally isolated from *D. nerii* larvae in Nanchang, Jiangxi, China, is preserved in our laboratory. DnCPV-23 was propagated by inoculating healthy *D. nerii* larvae collected from Nanchang, Jiangxi, China and fed clean *Nerium oleander* leaves.

The Sf9 cell line (Invitrogen, Carlsbad, CA, USA) was used to generate recombinant baculoviruses. To collect baculoviruses with recombinant proteins, 8 × 10^5^ Sf9 cells were added to glass-bottomed culture dishes and then transfected with 3 µg recombinant bacmids using Cellfectin^®^ Reagent (Invitrogen). At 72 h post-transfection, the supernatant was collected and centrifuged at 500× *g* for 5 min to remove cell debris. Harvested recombinant viruses were transferred to a sterile centrifuge tube and stored at 4 °C in darkness. The QB-MS Ms2-2 cell line, which originated from *Manduca sexta* eggs [34], was kindly provided by Prof. Changyou Li at Qingdao Agricultural University. This cell line has been employed for RNA interference, overexpression, and assays examining virus entry, attachment and internalization. Cell lines were cultured at 27 °C in Grace’s Insect Medium (Thermo Fisher Scientific, Waltham, MA, USA) supplemented with 10% (*v*/*v*) fetal bovine serum (FBS, ExCell Bio, Suzhou, China).

Polyclonal antibodies against DnCPV-23 virions, DnCPV-23 polyhedrin (PH), and virus attachment proteins (VAPs, including VP2 encoded by *S3*, VP3 encoded by *S4*, and VP5 encoded by *S8*) were prepared and preserved in our laboratory [35]. Rabbit IgG, anti-glutathione S-transferase (GST) polyclonal antibodies (pAbs), anti-Flag pAbs, and goat anti-rabbit horseradish peroxidase (HRP)-conjugated antibodies were purchased from Proteintech, Wuhan, China. Anti-MsAGL pAbs were prepared by Abclonal Technology (Wuhan, China).

### 2.2. Plasmids and Recombinant Proteins

To produce structural proteins VP2, VP3, VP4, VP5, and VP6, genomic segments *S3*, *S4*, *S5*, *S7*, and *S8* were amplified by PCR using a previously constructed cDNA library [3] and specific PCR primer pairs (Sangon, Shanghai, China) (Table 1). Recombinant C-terminal GST fusion vectors pFastBac 1^VP2-GST^, pFastBac 1^VP3-GST^, pFastBac 1^VP4-GST^, pFastBac 1^VP5-GST^ and pFastBac 1^VP6-GST^ were generated by cloning the amplified *S3*, *S4*, *S5*, *S7* and *S8* segments into the pFastBac™ 1 vector (Invitrogen). The sequence encoding the GST tag was cloned from pGEX-6P-1 (GE Healthcare, Piscataway, NJ, USA).

Sf9 cells were transfected with recombinant pFastBac™ 1 plasmids to generate recombinant baculoviruses vAc^VP2-GST^-ph, vAc^VP3-GST^-ph, vAc^VP4-GST^-ph, vAc^VP5-GST^-ph, and vAc^VP6-GST^-ph, which contain GST-tagged recombinant proteins. Cells were lysed using Cell Lysis Buffer for Western and IP (Beyotime, Shanghai, China), with the resulting lysates containing soluble fusion proteins purified using BeyoGold™ GST-tag Purification Resin (Beyotime). Protein concentrations were determined using a Bradford Protein Assay Kit (Beyotime). The estimated sizes of GST, VP2-GST, VP3-GST, VP4-GST, VP5-GST, and VP6-GST were 26, 148, 114, 98, 77, and 70 kDa, respectively (Supplementary Figure S1A).

*MsAGL* (GenBank Accession No.: JH668294.1) was amplified by PCR using gene-specific primers (Table 1) and QB-Ms2-2 cDNA reverse transcribed from total RNA extracted from QB-Ms2-2 cells. Constructs carrying the amplified fragments were prepared for the subsequent production of fusion proteins with a 6×His-tag at the N-terminal and a 3×Flag-tag at the C-terminal. Constructs were inserted into the pFastBac™ 1 vector using a ClonExpress^®^ II Ultra One Step Cloning Kit (Vazyme, Nanjing, China) to generate recombinant vectors pFastBac 1^His-MsAGL−3Flag^. Verified recombinant vectors were inserted into *Escherichia coli* DH10Bac competent cells to generate the corresponding bacmid bAc*^His-MsAGL−3Flag^*-ph. The estimated size of recombinant His-MsAGL-3Flag was 67 kDa. A sodium dodecyl sulfate-polyacrylamide gel electrophoresis (SDS-PAGE) analysis indicated that purified recombinant proteins were the expected sizes (Supplementary Figure S1B).

Based on structural and functional information on Uniprot (https://www.uniprot.org/uniprotkb/A0A922CEJ9/entry, accessed on 10 October 2025), a sequence encoding the soluble ectodomain of MsAGL (amino acids 144–603) and a C-terminal GST tag (MsAGL-EXR-GST) was inserted into the pFastBac™ 1 vector to generate recombinant vectors pFastBac 1^MsAGL-EXR-GST^. This region corresponds to the predicted extracellular domain, excluding the N-terminal signal peptide and transmembrane region. This design enabled soluble expression and purification of a recombinant protein that retains the functional receptor domain, which is essential for preserving the virus-interacting interface while ensuring protein solubility. Sf9 cells were transfected with the recombinant vector and then induced using IPTG to produce recombinant proteins. Cells were lysed using Cell Lysis Buffer for Western and IP (Beyotime), after which soluble fusion proteins were purified using BeyoGold™ GST-tag Purification Resin (Beyotime). According to an SDS-PAGE analysis, purified recombinant proteins were in accordance with the predicted size of MsAGL-EXR-GST (i.e., 77 kDa) (Supplementary Figure S1C).

### 2.3. Purification of DnCPV-23 Virions

To release DnCPV-23 virions from polyhedra, purified polyhedra were digested in a 0.2 M Na_2_CO_3_-NaHCO_3_ (pH 10.8) solution to obtain a clear lysate. After adjusting the lysate pH to 7.5 using 1 M Tris-HCl (pH 6.8), DnCPV-23 virions were purified via a linear 20% to 60% (*w*/*v*) sucrose gradient centrifugation [35]. The purified DnCPV-23 virion concentration was calculated on the basis of absorbance at 280 nm, which was determined using a UV-31000PC spectrophotometer (Mapada, Shanghai, China).

### 2.4. Preparation of Host Brush Border Membrane Vesicles (HBBMVs)

The midguts of 100 healthy fourth instar *D. nerii* larvae were collected to prepare HBBMVs as previously described [30,31]. Briefly, *D. nerii* larval BBMVs were carefully removed from the midgut. They were subsequently ground and resuspended in MET buffer (0.3 M mannitol, 17 mM Tris-HCl, and 5 mM EGTA, pH 7.5), after which an equal volume of 24 mM MgCl_2_ was added. The homogenate was mixed thoroughly, incubated on ice for 15 min, and centrifuged at 1900× *g* for 15 min at 4 °C. The supernatant was collected and centrifuged at 30,000× *g* for 30 min at 4 °C. The supernatant was discarded and the pellet was resuspended with MET buffer and 24 mM MgCl_2_ (1:1, *v*/*v*). The centrifugation steps were repeated twice, and then the final pellet was resuspended in 5 mL MET buffer. The extracted HBBMV concentration was determined using a Bradford Protein Assay Kit (Beyotime), after which HBBMVs were stored at −80 °C.

### 2.5. Characterization of the Attachment of DnCPV-23 Virions and VAPs to HBBMVs

Virion attachment assays were performed as previously described [31]. Briefly, purified DnCPV-23 virions were diluted to 0.01–100 µg. ELISA plates (96 wells) (Sangon) were pre-coated with HBBMVs (10 µg/mL) or BSA (10 µg/mL, Sangon) and blocked with 3% (*w*/*v*) BSA. 100 µL serially diluted purified DnCPV-23 virions or BSA (control) were added to wells. After 1 h incubation at 4 °C, the wells were washed three times using phosphate-buffered saline (PBS) containing 0.05% (*v*/*v*) Tween-20 (PBST). Anti-DnCPV-23 pAbs were added to wells, which were then incubated before the HRP-conjugated goat anti-rabbit antibody (Proteintech) was added. TMB reagent (Beyotime) was used for color development, with reactions terminated by adding 2 M H_2_SO_4_. The attachment of DnCPV-23 virions or BSA to HBBMVs was determined according to the OD_450_ value measured using a Multiskan FC Microplate Photometer (Thermo Fisher Scientific). DnCPV-23 virion–HBBMV binding rates were transformed to arcsine square root values. The significance of the differences among treatments was assessed by an ANOVA followed by a least square deviation (LSD) *t*-test in R (version 3.6.1).

### 2.6. Inhibition of the Attachment of DnCPV-23 Virions to HBBMVs by Anti-VAP Antibodies

DnCPV-23 virions (10 µg) were mixed with rabbit pre-immune serum (control), anti-DnVP2, anti-DnVP3, anti-DnVP4, anti-DnVP5, anti-VP6, or a combination of anti-DnVP2, anti-DnVP3, anti-DnVP4, anti-DnVP5, and anti-VP6 antibodies at specific fold dilutions. 96-wells ELISA plates (Sangon) were pre-coated with HBBMVs that had been diluted to 10 µg/mL and then blocked with 5% (*w*/*v*) BSA. Treated DnCPV-23 virions were added to HBBMV-coated wells and incubated. Virion attachment to HBBMVs was measured as described above. This analysis was repeated three times. The binding of DnCPV-23 virions to HBBMVs was determined according to the ratio of the OD_450_ value of DnCPV-23 bound to HBBMVs treated with anti-VAP antibodies to that of DnCPV-23 bound to HBBMVs treated with rabbit pre-immune serum. The average binding of DnCPV-23 virions to HBBMVs treated with rabbit pre-immune serum was set as 100%. The OD_450_ value for the binding of rabbit pre-immune serum, which was considered to be non-specific, was subtracted from the OD_450_ values for the binding of DnCPV-23 virions to HBBMVs. Binding rates were transformed to arcsine square root values. The significance of the difference between treatments and the control was assessed by a one-sample *t*-test in R (version 3.6.1).

### 2.7. Far-Western Blotting

A far-Western blotting assay was conducted according to a published method that was modified [31]. Briefly, HBBMVs (10 µg/lane) were separated in a 10% (*v*/*v*) SDS-PAGE gel and then proteins were transferred to a polyvinylidene difluoride (PVDF) membrane (Millipore, Billerica, MA, USA). The PVDF membrane was immersed in a PBS solution containing 5% (*w*/*v*) BSA for 2 h at room temperature to block non-specific protein-binding sites. The membrane was immersed in a solution comprising 10 µg/mL purified DnVP3-GST or GST alone (negative control) for 2 h at room temperature. Proteins that did not bind to the membrane were removed by washing three times with PBST. Next, rabbit anti-GST pAbs (Proteintech) were added, after which the membrane was incubated at room temperature for 3 h. Unconjugated antibodies were removed by washing three times with PBST. The membrane was incubated with HRP-conjugated goat anti-rabbit antibody (Proteintech) for 1.5 h and then washed three times with PBST. Chemiluminescence signals were detected using a BeyoECL Star Kit (Beyotime) and photographed using a ChemiScope 3600 Mini Chemiluminescence Imaging System (Clinx, Shanghai, China).

### 2.8. Liquid Chromatography-Tandem Mass Spectrometry (LC-MS/MS) Analysis

Target protein bands on the SDS-PAGE gel identified by far-Western blotting were carefully excised and then digested overnight using trypsin at 37 °C. The resulting peptides were desalted and concentrated for an LC-MS/MS analysis, which was performed using a TripleTOF 5600 system (SCIEX, Framingham, MA, USA). Peptide samples were trapped using a C18 Trap Column (5 µm, 5 × 0.3 mm, Eksigent, Framingham, MA, USA) and then diluted. Peptides were separated using an analytical column (75 µm × 150 mm, 3 µm particle size, 100 Å pore size, Eksigent), with 60 min analytical gradients established with two mobile phases (mobile phase A: H_2_O, 0.1% formic acid; mobile phase B: acetonitrile, 0.1% formic acid). The flow rate was set to 300 nl/min. Each scanning cycle contained one MS full scan (*m*/*z* = 350–1500, ion accumulation time = 250 ms) followed by 40 MS/MS scans (*m*/*z* = 1000–1500, ion accumulation time = 50 ms). Data generated by the TripleTOF 5600 system were analyzed using ProteinPilot (version 4.5) and the Paragon algorithm in the UniProt database. Data below a certain threshold (unused ≤1.3) were discarded. Host proteins bound to GST were considered background proteins, which were eliminated.

### 2.9. Co-Immunoprecipitation (Co-IP) and GST Pull-Down Assays

Dynabeads™ Protein G (Thermo Scientific) (50 µL) was incubated with 10 µg anti-GST at 4 °C for 8 h. Sf9 cells were co-infected with recombinant baculoviruses expressing prey (GST or recombinant VP3-GST protein) and bait (recombinant His-MsAGL-3Flag protein) proteins for 72 h. Sf9 cells were harvested and washed three times with PBS before being lysed in 200 µL cell lysis buffer (Beyotime) for 1 h at 4 °C. Cell lysates containing prey and bait proteins were collected and added to antibody-conjugated beads, which was followed by a 2 h incubation at room temperature. To remove unbound proteins, beads were washed three times with PBST. Washed beads were resuspended in 20 µL PBS and 5× loading buffer (Yeasen, Shanghai, China) and then heated in a boiling water bath for 10 min. Samples were loaded on a 10% SDS-PAGE gel for a Western blot analysis.

To perform a GST pull-down assay, the recombinant His-MsAGL-3Flag protein was expressed in Sf9 cells (Supplementary Figure S1B). His-MsAGL-3Flag (10 µg) was incubated with purified DnVP3-GST (20 µg) or GST (20 µg) at 4 °C for 2 h. Next, 50 µL GST-conjugated Beaver Beads™ GSH (Beaver, Suzhou, China) was added to the protein mixture, which was then incubated at 4 °C for 1 h. Beads were washed three times with PBST to remove the unbound protein complex. Beads were resuspended in 20 µL PBS and 5× loading buffer (Yeasen) and then heated in a boiling water bath for 10 min. Samples were then separated in a 10% SDS-PAGE gel for further analyses.

### 2.10. RNA Interference and Overexpression Assay

To conduct an RNA interference assay, QB-Ms2-2 cells were maintained at 27 °C in Grace’s Insect Medium (Thermo Fisher Scientific). For transfection, cells were seeded into 6-well plates at a density of 5 × 10^5^ cells per well and incubated overnight until they reached 70–80% confluence. For transfection, 80 nM siRNA was mixed with Cellfectin^®^ II Reagent in serum-free medium according to the manufacturer’s protocol. The mixture was incubated at room temperature for 20 min to form complexes. After 5–6 h of incubation at 27 °C, the transfection mixture was replaced with fresh complete medium. Transfected cells were incubated at 27 °C for 72 h to efficiently decrease the mRNA level of target genes. The silencing efficiency was confirmed by qPCR analysis. Details regarding primer sequences for the siRNA-based decrease in MsAGL expression are listed in Table 1. The cells transfected with siGFP were regarded as control (siControl).

For overexpression assay, QB-Ms2-2 cells were maintained at 27 °C in Grace’s Insect Medium (Thermo Fisher Scientific). For transfection, cells were seeded into 6-well plates at a density of 1 × 10^6^ cells per well. Cells in each well were transfected with 3.0 µg of the recombinant plasmid pIZ-His/V5-MsAGL (containing the full-length *MsAGL* gene) or the empty pIZ-His/V5 plasmid (control) using 4.0 µL of Cellfectin^®^ II Reagent (Invitrogen), according to the manufacturer’s protocol. Transfected cells were incubated at 27 °C for 48 h to ensure the target gene was overexpressed. Overexpression of the MsAGL protein was verified by Western blot using anti-MsAGL antibody.

### 2.11. Attachment, Internalization, and Infection Assays Involving DnCPV Virions and Cells

For a virus attachment assay, QB-Ms2-2 cells were incubated with DnCPV-23 virions at a specific multiplicity of infection (MOI) at 4 °C for 1 h. To remove unattached virions, cells were washed three times with cold PBS. Washed cells were collected for further analyses. The experiments were performed in triplicates. Protein levels of PH and MsAGL were quantified by measuring band intensities on Western blots using ImageJ 1.54p and normalized to GAPDH.

To conduct a virus internalization assay, QB-Ms2-2 cells were incubated with DnCPV-23 virions at a specific MOI at 4 °C for 1 h. Cells were washed three times with cold PBS and then incubated at 27 °C for 1 h. Virions that were not internalized were removed by washing cells three times with cold PBS. Washed cells were collected for further analyses. The experiments were performed in triplicates. Protein levels of PH and MsAGL were quantified by measuring band intensities on Western blots using ImageJ 1.54p and normalized to GAPDH.

To perform a virus infection assay, QB-Ms2-2 cells or Sf9 cells were incubated with DnCPV-23 virions at a specific MOI at 27 °C for 6 h. The medium was replaced with fresh Grace’s Insect Medium (Thermo Fisher Scientific) supplemented with 10% (*v*/*v*) FBS (ExCell Bio). Cells were cultured for specific durations and then harvested for further analyses. The experiments were performed in triplicates. Protein levels of PH and MsAGL were quantified by measuring band intensities on Western blots using ImageJ 1.54p and normalized to GAPDH.

### 2.12. Real-Time Quantitative PCR (RT-qPCR)

An RT-qPCR analysis was completed to quantify the mRNA level of target genes. Total RNA was extracted from QB-Ms2-2 cells using TRIzol reagent (Life Technologies, Carlsbad, CA, USA), after which first-strand cDNA was synthesized using a HiScript III 1st Strand cDNA Synthesis Kit (Vazyme, China). The 20 µL RT-qPCR mixture contained 10 µL 2× ChamQ SYBR qPCR Master Mix (Vazyme, Nanjing, China), 7.4 µL ddH_2_O, 1 µL cDNA template, and 0.8 µL forward and reverse primers (Table 1). Three replicates were prepared per gene. A Quant Studio™ 7 Flex Real-Time PCR System (Applied Biosystems, Waltham, MA, USA) and the following program were used for the RT-qPCR analysis: 95 °C for 10 s; 40 cycles of 95 °C for 15 s, 60 °C for 30 s, and 72 °C for 30 s. One cycle was added for a melting curve analysis for all reactions. The 2^−ΔΔCt^ method was used to quantify both the *S10* level in the viral attachment assay and the *S10* mRNA levels in the viral internalization, entry, and infection assays [36], with *GAPDH* (Genbank accession: XM_030173465.2) selected as a reference gene [37,38].

### 2.13. MsAGL Blocking and Virus Neutralization Bioassays

To complete an MsAGL blocking assay, 6-well plates were pre-coated with Sf9 cells. The purified extracellular region of MsAGL (MsAGL-EXR) and GST at specific concentrations were separately mixed with DnCPV-23 virions (MOI = 1) and incubated at 4 °C for 2 h. The virus–protein mixture was incubated with Sf9 cells at 27 °C for 4 h. The medium was replaced with fresh Grace’s Insect Medium (Thermo Fisher Scientific) supplemented with 10% (*v*/*v*) FBS (ExCell Bio), after which cells were incubated at 27 °C for 48 h before being harvested for further analyses. The experiments were performed in triplicates. *S10* mRNA levels were calculated using the 2^−ΔΔCt^ method, with *GAPDH* (Genbank accession: XM_030173465.2) selected as a reference gene. Protein levels of PH were quantified by measuring band intensities on Western blots using ImageJ 1.54p and normalized to GAPDH.

To conduct a virus neutralization bioassay, third instar *D. nerii* larvae were starved for 16 h before being treated. DnCPV-23 virions (MOI = 10) were pre-incubated with PBS BSA (10 µg/mL) or MsAGL (10 µg/mL) at room temperature for 1 h. Larvae fed BSA (10 µg/mL) served as the negative control (CK), whereas larvae treated with untreated DnCPV-23 virions (MOI = 10) were used as the positive control. DnCPV-23 virions or BSA were spread evenly on the surface of fresh *N. oleander* leaves, which were then air-dried before being fed to larvae. All larvae used in the bioassay were incubated at 27 ± 1 °C, with 65% ± 5% relative humidity and a 16 h light:8 h dark cycle. Each treatment group contained 24 larvae, and the bioassay was performed twice. Larval mortality was recorded daily until all individuals had either died or pupated. Survival was analyzed using the Kaplan–Meier method and the “survival” package in R (version 3.6.1). The significance of the differences in survival curves among treatments was determined using a log-rank test [39].

## 3. Results

### 3.1. VP3 Mediates the Binding of DnCPV-23 to HBBMVs

**Figure 1:**
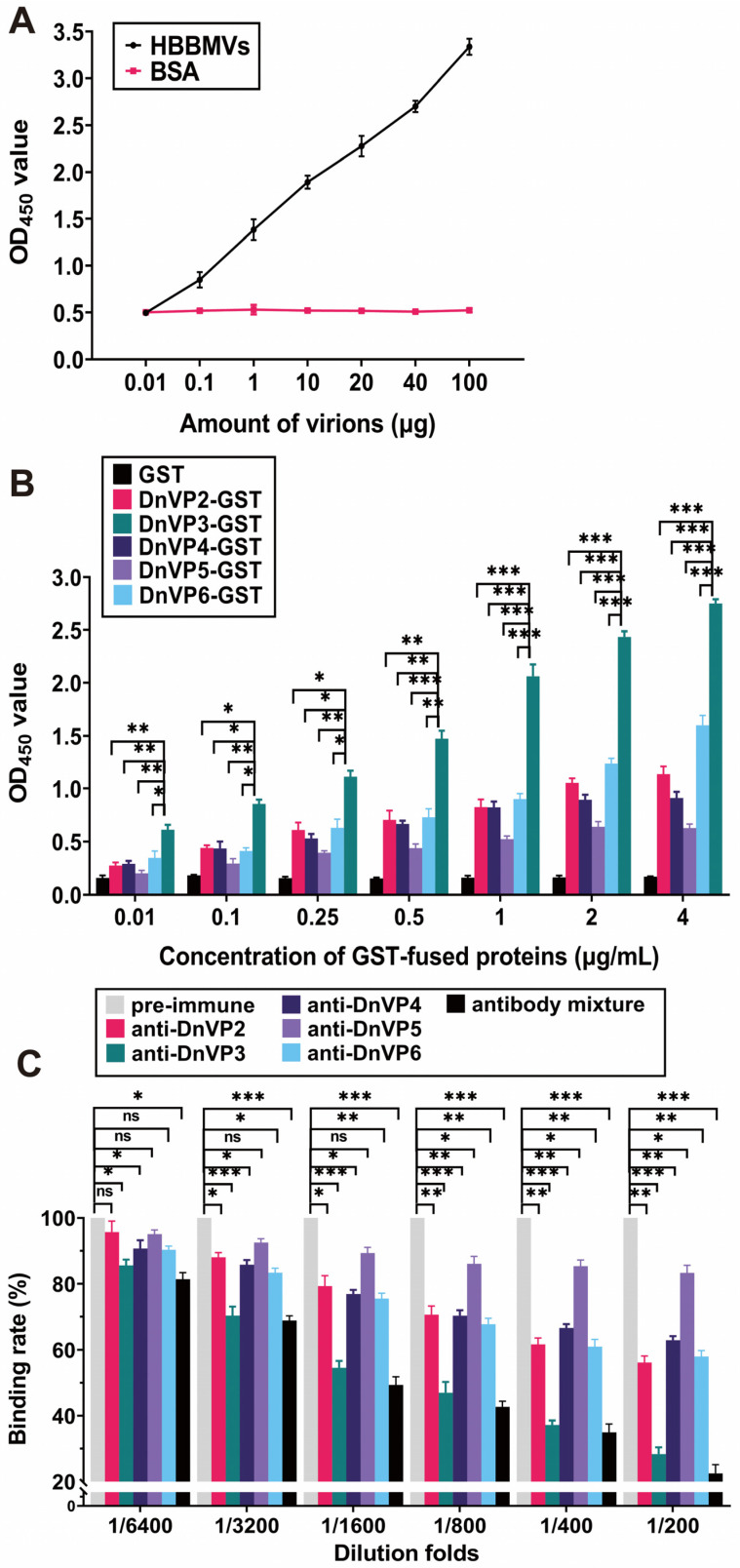
VP3 mediates DnCPV-23 binding to HBBMVs. (**A**) Binding of DnCPV-23 virions to HBBMVs. Attachment of virions to BSA (control) or HBBMVs was determined according to OD_450_ values by ELISA. Data are presented as the mean ± SEM of triplicates; (**B**) Binding of GST-tagged viral structural proteins to HBBMVs. Binding levels were determined according to OD_450_ values. Each bar represents the mean ± SEM of triplicates; (**C**) Inhibition of viral attachment via antibodies targeting viral structural proteins. HBBMVs were incubated with pre-immune serum antibodies (control), antibodies against viral structural proteins, or the corresponding antibody mixture prior to treatment with DnCPV-23 virions. The antibody mixture was prepared by mixing equal proportions of antibodies against viral structural proteins and then diluted to the indicated folds. The binding rate of DnCPV-23 virions treated with pre-immune rabbit serum was defined as 100%. *: *p* < 0.05; **: *p* < 0.01; ***: *p* < 0.001; ns: not significant, *p* > 0.05.

The attachment of DnCPV-23 virions to HBBMVs was examined by coating ELISA plates with HBBMVs and then adding serially diluted purified virions or BSA. A concentration-dependent binding of DnCPV-23 to HBBMVs was observed (Figure 1A), whereas no specific attachment was observed when BSA was incubated with HBBMVs. Therefore, the purified DnCPV-23 virions were suitable for further analyses.

To determine whether recombinant viral structural proteins could attach to HBBMVs, plate wells were pre-coated with HBBMVs, and then purified GST, BSA (control), and GST-tagged candidate VAPs, including DnVP2-GST, DnVP3-GST, DnVP4-GST, DnVP5-GST, and DnVP6-GST, were added (Figure 1B and Supplementary Figure S1A). DnVP2-GST, DnVP3-GST, DnVP4-GST, DnVP5-GST and DnVP6-GST were all revealed to bind to HBBMVs in a dose-dependent manner, whereas binding was undetectable between GST and HBBMVs. Compared with the other GST-tagged candidate VAPs, DnVP3-GST bound to HBBMVs significantly more strongly, suggesting that VP3 is the major VAP. To validate this finding, DnCPV-23 virions were pre-incubated with serially diluted anti-DnVP2, anti-DnVP3, and anti-DnVP5 alone or a mixture of these three antibodies. Antibody-treated DnCPV-23 virions were incubated with HBBMVs and then the amount of attached DnCPV-23 virions was determined (Figure 1C). Compared with the rabbit pre-immune serum, DnCPV-23 virions treated with anti-DnVP2, anti-DnVP3, anti-DnVP4, or anti-DnVP6 antibodies at 1:3200 dilution significantly decreased viral attachment to HBBMVs. At 1:200 dilution, anti-DnVP2, anti-DnVP3, anti-DnVP4, and anti-DnVP6 decreased the binding of DnCPV-23 to HBBMVs to 56.15% ± 2.82%, 28.35% ± 2.94%, 62.86% ± 1.76%, and 57.97% ± 2.61%, respectively. Anti-DnVP3 had the strongest inhibitory effect among the antibodies developed for candidate VAPs, reflecting the importance of VP3 for viral attachment. Moreover, a 1:200 dilution of the antibody mixture decreased the binding rate to 22.51% ± 3.77%. Notably, a 1:6400 dilution of the antibody mixture significantly decreased the binding rate to 81.41% ± 2.83% (Figure 1C), suggesting that in addition to VP3, other candidate VAPs contribute to viral attachment.

**Figure 2:**
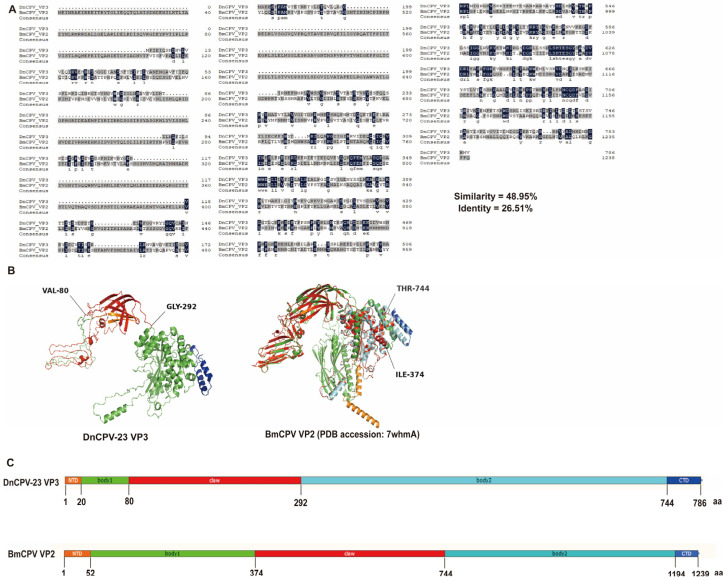
Structural homology analysis of DnCPV-23 VP3 with BmCPV VP2. (**A**) Sequence alignment showing identity and similarity percentages; (**B**) Predicted secondary structure of VP3, modeled on BmCPV VP2, showing the N-terminal domain (NTD, orange), body 1 domain (green), claw domain (red), body 2 domain (cyan), and C-terminal domain (CTD, blue); (**C**) Predicted domain organization of VP3, inferred from the VP2 template, illustrating the same domain arrangement: NTD (orange), body 1 (green), claw (red), body 2 (cyan), and CTD (blue).

The VP3 secondary structure was predicted using the Phyre2.2 server (https://www.sbg.bio.ic.ac.uk/phyre2, accessed on 15 September 2025) [40]. A comparison of amino acid sequences revealed 26.51% sequence identity and 48.95% sequence similarity between DnCPV-23 VP3 and the BmCPV spike protein VP2 (PDB: 7WHMA), which is encoded by *S3* (Figure 2A). A homology model was constructed for DnCPV-23 VP3 using the VP2 structure as a template (Figure 2B,C). On the basis of these analyses, we speculated that DnCPV-23 VP3 is a spike protein responsible for mediating viral attachment to midgut proteins in *D. nerii*.

### 3.2. Identification of HBBMV Proteins That Interact with DnCPV-23 VP3

**Figure 3:**
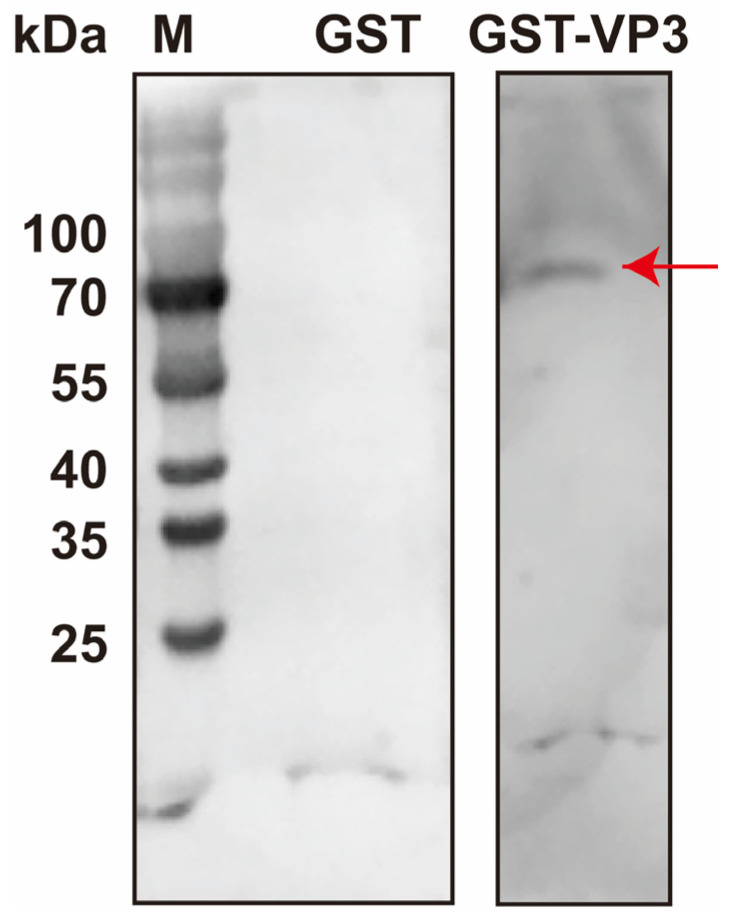
Identification of HBBMV proteins that interact with VP3 on the basis of far-Western blotting. HBBMV proteins were separated in a 10% SDS-PAGE gel and probed with purified GST-VP3 or GST alone. Lane M: protein molecular mass marker. The red arrow in lane 3 indicates HBBMV proteins that interact with VP3.

The above-mentioned results indicated that DnCPV-23 binds to HBBMVs via VP3. To identify HBBMV proteins directly involved in viral attachment, a far-Western blot analysis was performed. A distinct band (approximately 70 kDa) was detected on a PVDF membrane overlaid with VP3-GST, whereas clear bands were undetectable on the GST-overlaid PVDF membrane, confirming that GST alone did not interfere with the binding of VP3 to HBBMVs (Figure 3). The 70 kDa band was subsequently subjected to LC-MS/MS analyses to identify HBBMV proteins that interact with VP3. Considering the lack of a comprehensively annotated *D. nerii* genome, a protein database for the closely related species *Manduca sexta* was used as a reference database. LC-MS/MS results are provided in Table 2. According to Gene Ontology annotations (https://www.uniprot.org/uniprotkb/A0A922CEJ9/entry#subcellular_location, accessed on 25 October 2025), *M. sexta* alpha-glucosidase (UniProt Accession No.: A0A922CEJ9, MsAGL) is localized to the apical plasma membrane of host midgut epithelial cells, where BBMVs are presented, and MsAGL had a molecular weight that was consistent with the observed 70 kDa band [41]. Therefore, MsAGL may be involved in the attachment of DnCPV-23 to midgut cells.

### 3.3. MsAGL Interacts with DnCPV-23 VP3

**Figure 4:**
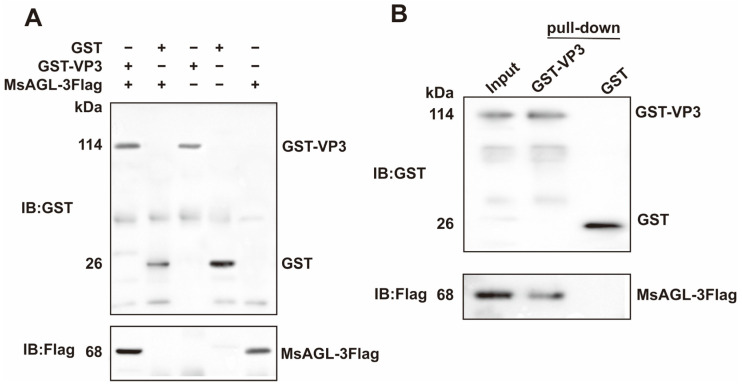
Interaction between MsAGL and VP3. (**A**) Co-immunoprecipitation analysis. Immunoprecipitated complexes (lanes 1–3) along with GST (lane 4) and MsAGL (lane 5) protein controls were separated by SDS-PAGE, transferred to a PVDF membrane, and probed using anti-GST or anti-Flag pAbs; (**B**) GST pull-down assay. Input lysates containing GST-VP3 and MsAGL-3Flag (lane 1) and proteins pulled down by GST-VP3 (lane 2) or GST alone (lane 3) were analyzed by immunoblotting with anti-GST or anti-Flag pAbs.

Co-IP assays were completed to confirm that DnVP3 can interact with MsAGL in vivo. Anti-Flag pAbs were used to immunoprecipitate the prey–bait protein complex on beads and detect the prey protein MsAGL (Supplementary Figure S1B). Anti-GST pAbs were used to determine whether DnVP3 or GST can interact with MsAGL. According to assay results, DnVP3 interacted with MsAGL, but GST did not (Figure 4A), indicating that the GST tag did not influence the interaction between DnVP3 and MsAGL. The DnVP3–MsAGL interaction in vitro was validated by conducting GST pull-down assays. MsAGL-3Flag was incubated with DnVP3-GST or GST prior to incubating with GST-conjugated beads. The protein mixture bound to the beads were analyzed by immunoblotting using anti-GST pAbs and anti-Flag pAbs. The input consisted of the mixture containing GST-VP3 and MsAGL-3Flag prior to bead incubation. Anti-GST immunoblotting confirmed the presence of GST-VP3 and GST on the beads, while anti-Flag immunoblotting revealed that MsAGL-3Flag specifically interacted with GST-VP3 but not with GST alone, implying that the GST tag did not interfere with the protein interaction (Figure 4B). Thus, Co-IP and GST pull-down assays verified the specific interaction between DnVP3 and MsAGL, which may mediate the attachment of DnCPV-23 to host cells.

### 3.4. MsAGL Mediates DnCPV-23 Attachment and Internalization

**Figure 5:**
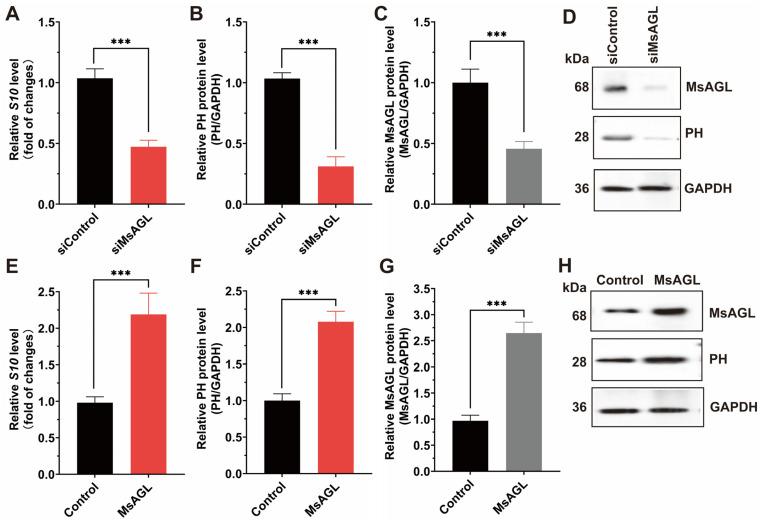
Role of MsAGL in DnCPV-23 attachment. The effects of MsAGL knockdown and overexpression on viral attachment were assessed by measuring the viral gene *S10* (encoding the capsid protein PH) levels and corresponding protein levels at 1 h post-infection (h p.i.). The time point designated as h p.i. corresponds to the duration of virus–cell interaction. (**A**,**E**) Relative *S10* gene levels following MsAGL knockdown (**A**) or overexpression (**E**) in QB-MS 2-2 cells, indicating the number of virions that attached to the cells. (**B**,**F**) Corresponding relative PH protein levels after MsAGL knockdown (**B**) or overexpression (**F**), reflecting the viral attachment level. (**C**,**G**) Validation of MsAGL expression after knockdown (**C**) or overexpression (**G**). (**D**,**H**) Western blot analysis confirming protein levels of MsAGL, PH, and GAPDH under knockdown (**D**) or overexpression (**H**) conditions. Data are shown as mean ± SEM from three independent experiments, each performed in triplicate. ***: *p <* 0.001.

Considering MsAGL was localized to the apical plasma membrane of host midgut epithelial cells, which are the primary targets of DnCPV-23, and MsAGL can interact with DnCPV-23, we hypothesized that MsAGL was involved in DnCPV-23 attachment and internalization into host cells. To determine whether MsAGL facilitates DnCPV-23 attachment during cell entry, we performed viral attachment assays under conditions that allow virion binding but not internalization. QB-Ms2-2 cells were pre-chilled to 4 °C, incubated with DnCPV-23 virions (MOI = 10) for 1 h to permit surface binding, and then thoroughly washed to remove unattached virions. Attached virons were quantified by measuring the *S10* gene level via RT-qPCR and viral capsid protein PH via Western blotting. Cells transfected with siRNA targeting MsAGL (siMsAGL) or control siRNA (siGFP, siControl) were subjected to the viral attachment assay. RT-qPCR and Western blot analysis revealed that *S10* gene level and PH protein levels were significantly decreased in MsAGL-silenced cells compared to controls (Figure 5A,B,D). Efficient MsAGL knockdown was confirmed at the protein level (Figure 5C,D). To test whether MsAGL is sufficient to promote viral attachment, we overexpressed MsAGL in QB-Ms2-2 cells prior to performing the attachment assay. RT-qPCR analysis showed that *S10* gene level and PH protein levels were significantly higher in MsAGL-overexpressing cells than in controls (cells transfected with the empty pIZ/His-V5 vector) (Figure 5E). Accordingly, Western blot detection of viral PH protein revealed a significant increase in the PH/GAPDH ratio upon MsAGL overexpression (Figure 5F,H). Overexpression efficiency was validated by elevated MsAGL protein levels, with quantification confirming a significant increase relative to controls (Figure 5G,H). Together, these results demonstrate that MsAGL is required for DnCPV-23 attachment to host cells.

**Figure 6:**
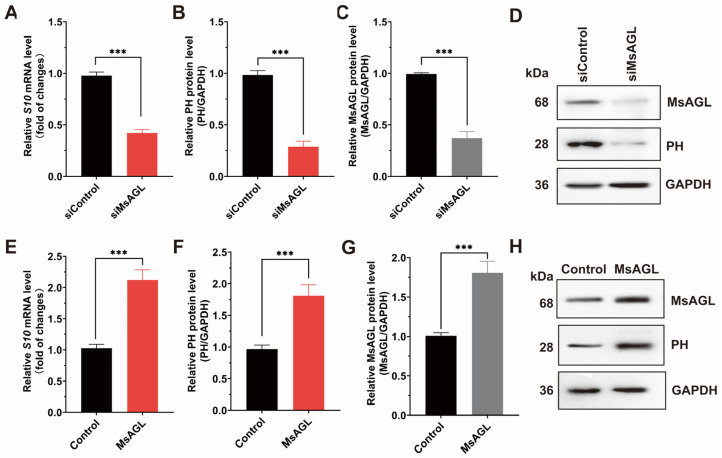
Role of MsAGL in DnCPV-23 internalization. The contribution of MsAGL to viral internalization was evaluated through changes in *S10*/PH expression after virus internalization in host cells at 2 h p.i. The time point designated as h p.i. corresponds to the duration of virus–cell interaction. (**A**,**E**) Relative *S10* (encoding PH) mRNA levels after MsAGL knockdown (**A**) or overexpression (**E**) in QB-Ms2-2 cells, representing the number of virions that internalized into host cells. (**B**,**F**) Relative PH protein levels following MsAGL knockdown (**B**) or overexpression (**F**). (**C**,**G**) MsAGL expression levels upon knockdown (**C**) or overexpression (**G**). (**D**,**H**) Western blot analysis of MsAGL, PH, and GAPDH after knockdown (**D**) or overexpression (**H**). Data are shown as mean ± SEM from three independent experiments, each performed in triplicate. ***: *p <* 0.001.

Following viral attachment, internalization is the next step during a viral infection. To investigate how MsAGL influences the internalization of DnCPV-23, we decreased MsAGL expression in QB-Ms2-2 cells by transfecting them with MsAGL-targeting siRNA. Transfected cells were then infected with DnCPV-23 virions (MOI = 5) at 4 °C to facilitate viral attachment, after which they were transferred to 27 °C to initiate internalization. The effect of decreased MsAGL expression on DnCPV-23 internalization was assessed by performing RT-qPCR and Western blot analyses. A comparison with control cells (cells transfected with siGFP) revealed that the mRNA and protein level of *S10* gene decreased in cells in which *MsAGL* expression was knocked down, indicating that decreasing *MsAGL* expression suppresses the internalization of DnCPV-23 by QB-Ms2-2 cells (Figure 6A–D). To clarify the role of MsAGL in DnCPV-23 internalization, we overexpressed MsAGL in QB-Ms2-2 cells, which were then infected with DnCPV-23 virions (MOI = 1). Changes in viral internalization due to MsAGL overexpression were determined by conducting RT-qPCR and Western blot analyses. Interestingly, MsAGL overexpression significantly enhanced DnCPV-23 internalization (compared with the internalization by control cells transfected with empty pIZ/His-V5 vector) (Figure 6E–H). Collectively, these findings indicate that MsAGL affects the internalization of DnCPV-23.

### 3.5. MsAGL Is Involved in DnCPV-23 Entry

**Figure 7:**
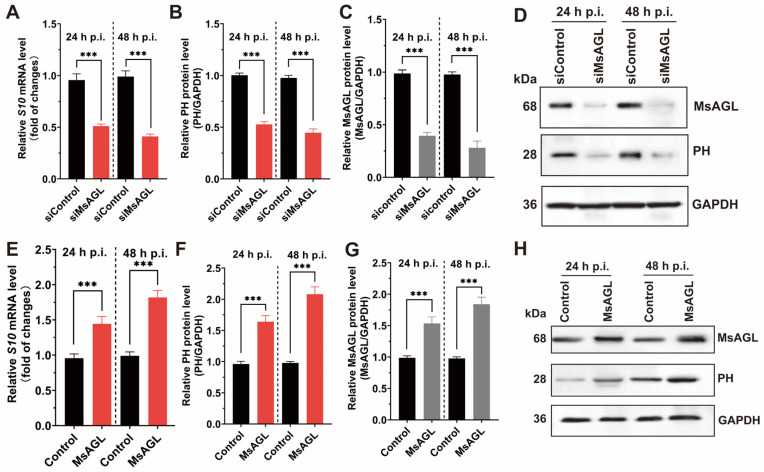
Role of MsAGL in DnCPV-23 entry. The effects of MsAGL knockdown and overexpression on viral attachment were assessed by measuring the expression of viral gene *S10* (encoding the capsid protein PH) and corresponding protein levels at 24 h p.i. and 48 h p.i. The time point designated h p.i. corresponds to the duration of virus–cell interaction. (**A**,**E**) Relative *S10* mRNA levels following MsAGL knockdown (**A**) or overexpression (**E**) in QB-Ms2-2 cells, indicating transcriptional activity during viral entry. (**B**,**F**) Corresponding relative PH protein levels after MsAGL knockdown (**B**) or overexpression (**F**), reflecting translational output. (**C**,**G**) Validation of MsAGL expression after knockdown (**C**) or overexpression (**G**). (**D**,**H**) Western blot analysis confirming protein levels of MsAGL, PH, and GAPDH under knockdown (**D**) or overexpression (**H**) conditions. Data are shown as mean ± SEM from three independent experiments, each performed in triplicate. ***: *p* < 0.001.

Viral entry into host cells is a multistep process that includes attachment, internalization, uncoating, and genome release. Given our previous findings that MsAGL is involved in both the attachment and internalization of DnCPV-23, we hypothesized that it plays a role in viral entry. This hypothesis was tested by analyzing the effect of decreased and increased MsAGL expression on DnCPV-23 entry into host cells. We used QB-MS Ms2-2 cells derived from *M. sexta* eggs as host cells. Viral entry was assessed by quantifying viral *S10* mRNA levels and capsid protein PH abundance at 24 and 48 h post-infection, reflecting the number of virions that had successfully entered host cells. After transfection with MsAGL-targeting siRNA, the cells were infected with DnCPV-23 virions (MOI = 5). The effect of decreased MsAGL level on DnCPV-23 entry into host cells was clarified on the basis of RT-qPCR and Western blotting. A comparison with control cells (cells transfected with siGFP, labeled as siControl) indicated that decreasing MsAGL expression significantly inhibited the entry of DnCPV-23 (Figure 7A–D). To further elucidate the role of MsAGL in DnCPV-23 entry, we overexpressed MsAGL in QB-Ms2-2 cells, which were then infected with DnCPV-23 virions (MOI = 1). The effect of MsAGL overexpression on DnCPV-23 entry was analyzed by conducting RT-qPCR and Western blot analyses. Notably, MsAGL overexpression significantly increased DnCPV-23 entry (relative to the control cells transfected with the empty pIZ/His-V5 vector, labeled as Control) (Figure 7E–H). Considered together, these results suggest that MsAGL contributes to DnCPV-23 entry into host cells.

### 3.6. Ectodomain of MsAGL Neutralizes DnCPV-23 Infections of Host Cells and D. nerii

**Figure 8:**
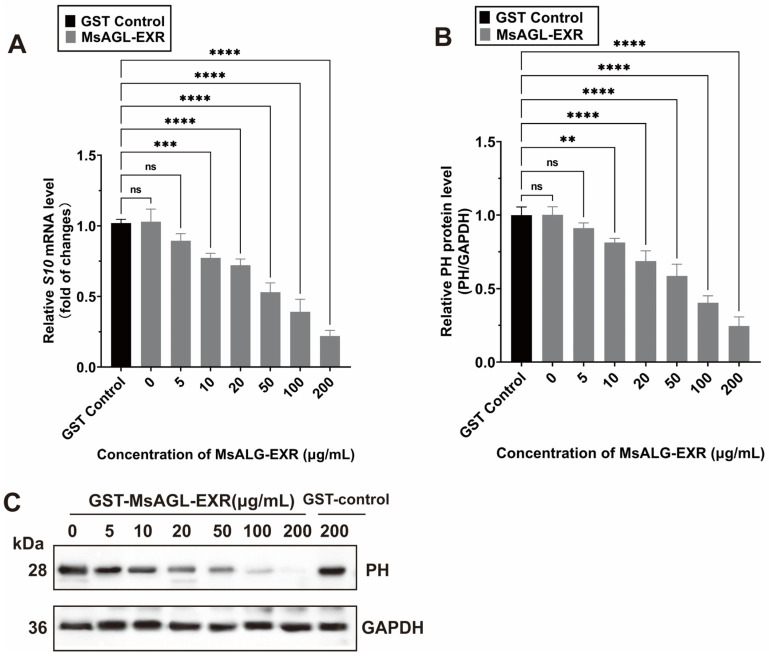
MsAGL neutralizes the infection of host cells by DnCPV-23. (**A**,**B**) Relative *S10* (encoding PH) mRNA level (**A**) and PH protein level (**B**) in Sf9 cells following a pre-incubation with MsAGL-EXR at 48 h p.i.; (**C**) Western blot analysis of the PH protein in Sf9 cells after the virus was pre-incubated with increasing concentrations of GST-MsAGL-EXR or GST alone at 48 h p.i. Data are shown as mean ± SEM from three independent experiments, each performed in triplicate.**: *p* < 0.01; ***: *p* < 0.001; ****: *p* < 0.0001; ns: not significant, *p* > 0.05.

The above-mentioned results for the *M. sexta*-derived cell line QB-Ms2-2 indicate that an *M. sexta* alpha-glucosidase is involved in DnCPV-23 entry, including attachment and internalization. Earlier research showed that DnCPV-23 can infect and replicate in *Spodoptera frugiperda* cell line Sf9 and *M. sexta* cell line QB-Ms2-2 [35,38]. To investigate whether MsAGL facilitates the entry of DnCPV-23 into other host cells, we performed in vitro blocking assays using DnCPV-23 virions and a recombinant MsAGL ectodomain (MsAGL-EXR) (Supplementary Figure S1C). Sf9 cells were infected with DnCPV-23 virions mixed with different concentrations of the purified GST-MsAGL-EXR protein or GST alone (Figure 8C). The Sf9 cells at 48 h p.i. were harvested for analyses. The subsequent analysis showed that GST-MsAGL-EXR inhibited the DnCPV-23 infection in a dose-dependent manner. More specifically, 10 µg/mL GST-MsAGL-EXR had a significant detrimental effect on infection, decreasing the *S10* mRNA level to 0.774 ± 0.026-fold of the control (Figure 8A) and the PH protein level to 0.814 ± 0.023-fold of the control (Figure 8B). Increasing the GST-MsAGL-EXR concentration to 200 µg/mL further decreased the *S10* mRNA level to 0.220 ± 0.033-fold of the control (Figure 8A) and the PH level to 0.244 ± 0.051-fold of the control (Figure 8B). By contrast, GST alone had no effect on the DnCPV-23 infection (Figure 8A,B). These results provide further evidence that MsAGL serves as an entry factor during an infection of host cells by DnCPV-23.

**Figure 9:**
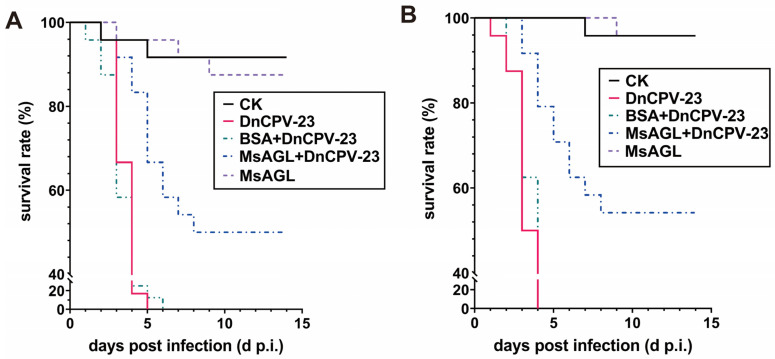
MsAGL neutralizes the infection of *D. nerii* larvae by DnCPV-23. (**A**,**B**) Survival rates of *D. nerii* larvae (n = 24 per group) in two independent bioassays. Larvae were fed DnCPV-23 virions (positive control, DnCPV), MsAGL alone (MsAGL), DnCPV-23 virions pre-treated with BSA (BSA + DnCPV-23),DnCPV-23 virions pre-treated with MsAGL (MsAGL + DnCPV-23) or BSA alone (CK).

DnCPV-23 was originally isolated from *D. nerii* [3]. To determine whether MsAGL can neutralize DnCPV-23 infections of its original host, in vivo neutralization assays were performed using *D. nerii* larvae. *D. nerii* larvae were fed BSA alone (CK), DnCPV-23 virions alone (DnCPV-23), DnCPV-23 virions pre-treated with 10 µg/mL MsAGL (MsAGL + DnCPV-23) or BSA (BSA + DnCPV-23). By 14 days post-infection, all *D. nerii* larvae died or pupated. In the first bioassay, the final survival rates of *D. nerii* larvae fed BSA alone, DnCPV-23 virions alone, virions pre-treated with BSA, and virions pre-treated with MsAGL were 91.7%, 0%, 0%, and 50.0%, respectively (*n* = 24) (Figure 9A). The survival curve of larvae fed DnCPV-23 virions differed significantly from that of larvae fed virions pre-treated with MsAGL (*χ*^2^ = 26.831, *p* < 0.001) (Table 3). The second bioassay yielded similar results. Specifically, the survival rates were 95.8%, 0%, 0%, and 54.2% for larvae fed BSA alone, DnCPV-23 virions alone, virions pre-treated with BSA, and virions pre-treated with MsAGL, respectively (*n* = 24) (Figure 9B). The survival curve of larvae fed virions alone differed significantly from that of larvae fed virions pre-treated with MsAGL (*χ*^2^ = 30.302, *p* < 0.001) (Table 4). These results indicate that MsAGL did not completely protect *D. nerii* from DnCPV-23, but it partially neutralized the infection in vivo. This suggests that additional unknown factors may be involved in facilitating viral entry into host cells.

As MsAGL not only inhibited the infection of Sf9 cells by DnCPV-23 virions under in vitro conditions but also suppressed DnCPV-23 infection in its host, *Daphnis nerii*. Through amino acid sequence alignment, MsAGL showed similarity to alpha-glucosidase proteins from *S. litura* (Uniprot accession: A0A9J7DWA6), *Trichoplusia ni* (Uniprot accession: A0A7E5VBV1), *S. frugiperda* (Uniprot accession: A0A9R0EUG7), *S. exigua* (Uniprot accession: A0A835GKK3), *B. mori* (Uniprot accession: A0A8R2G7K6), *H. armigera* (Uniprot accession: A0A2W1BLN8), and *G. mellonella* (Uniprot accession: A0ABM3MYL6) (Table 5). Therefore, it can be inferred that alpha-glucosidase may serve as a general entry factor involved in the cell entry of DnCPV-23 into multiple host cells.

## 4. Discussion

CPVs are important viral pathogens that can infect a wide range of insects, having been isolated from more than 250 insect species reared in laboratories or collected from fields [42]. In addition to the global horticultural pest *D. nerii*, other insect pests in the family Sphingidae are susceptible to DnCPV-23 [3]. However, research on DnCPV-23 infections of host cells has been hindered by the lack of an established *D. nerii* cell line. Consequently, *S. frugiperda* cell line Sf9 and *M. sexta* cell line QB-Ms2-2 can serve as alternative host cell lines for investigating DnCPV-23 infections [35,38].

Similar to infections by other CPVs, DnCPV-23 infections are restricted to the host digestive system, primarily the midgut. A successful viral infection is initiated by the attachment of virions to the host midgut epithelial cells. This is followed by the internalization of virions, which triggers the subsequent infection cascade, with viral spike proteins playing essential roles. In the thoroughly characterized MRV, surface spike protein σ1 serves as an attachment protein that mediates viral entry into host cells [5]. Spike protein VP4 has a similar role in RV [43]. In BTV, spike protein VP7 enables virions to attach to host cells [26]. For GCRV, outer capsid spike protein VP5 serves as an attachment protein for GCRV type I [44], whereas outer spike protein VP56 functions as an attachment protein for GCRV type II [45]. In DpCPV-1, VP3 and VP4 mediate viral attachment [31,46]. Cryo-electron microscopy-based examinations of BmCPV-1 identified VP2, which is encoded by genomic segment *S3*, as a spike protein; its atomic structure has been resolved in detail [47]. To comprehensively characterize how DnCPV-23 infects host cells, VAPs must be identified. In the current study, VP3 was observed to bind to HBBMVs significantly more strongly than other structural proteins. Additionally, anti-DnVP3 had the strongest inhibitory effects among antibodies targeting candidate VAPs. Moreover, the secondary structure of VP3 resembles that of BmCPV-1 VP2. Therefore, in DnCPV-23, VP3 encoded by genomic segment *S4* is a spike protein responsible for mediating viral attachment and internalization.

To successfully infect host cells, viral pathogens must attach to the host cell surface and bind to receptors on the cell membrane, which is also important for determining the viral host range and cellular tropism [5,6]. Considering DnCPV-23 infections are restricted to the host midgut, DnCPV-23 receptors are likely located on midgut columnar cell BBMVs [32]. Although DnCPV-23 was first isolated from *D. nerii*, the *D. nerii* genome has not been comprehensively annotated, which has limited the utility of the available genetic information for identifying receptors. By contrast, *M. sexta* has a thoroughly annotated genome and belongs to the same family (Sphingidae) as *D. nerii.* Moreover, DnCPV-23 can infect and replicate in *M. sexta* cell line QB-Ms2-2 [38]. Therefore, we screened the *M. sexta* protein database for candidate receptors that interact with the DnCPV-23 spike protein VP3. Far-Western blotting and LC/MS-MS data indicated that an alpha-glucosidase of *M. sexta* can interact with VP3. In vivo and in vitro interactions between this alpha-glucosidase and VP3 were confirmed by Co-IP and GST pull-down assays, respectively. Decreasing MsAGL expression significantly inhibited the attachment and internalization of DnCPV-23, whereas overexpressing MsAGL had the opposite effects. Furthermore, MsAGL was revealed to inhibit DnCPV-23 infections of host cells and neutralize DnCPV-23 infections of *D. nerii.* Accordingly, MsAGL serves as an entry factor during DnCPV-23 infections of host cells. Viral entry into host cells is a complex process that may involve multiple entry factors. Inhibiting one or a few of these factors may not completely prevent viral entry into host cells. For instance, the cell entry of enterovirus EV71 involves two receptors: human P-selectin glycoprotein ligand-1 (PSGL-1) and human scavenger receptor B2 (hSCARB2). Among these, hSCARB2 is expressed on most EV71-susceptible cells; however, neutralizing hSCARB2 on the surface of these cells only partially inhibits EV71 cell entry [48,49]. Therefore, simultaneously blocking multiple entry factors will more effectively inhibit viral cell entry.

Alpha-glucosidases are alpha-amylase family members that hydrolyze the glycosidic linkages of oligosaccharides. These enzymes are localized to the apical membrane of epithelial cells by a glycosylphosphatidylinositol anchor. In addition to aiding digestion, alpha-glucosidases may also contribute to virus recognition and invasion processes in several insects [50]. For example, in *Bemisia tabaci*, the alpha-glucosidase gene *Bta11975* is highly expressed during virus acquisition. The encoded enzyme is highly abundant in salivary glands and the gut. Suppressed *Bta11975* expression due to RNA interference adversely affects the ability of *B. tabaci* to acquire and transmit Tomato chlorosis virus, with viral load positively correlated with gene expression levels [51]. Interestingly, inhibited alpha-glucosidase activities positively modulate the resistance of *Culex quinquefasciatus* to a *Bacillus sphaericus* toxin [52]. Another study showed that an alpha-glucosidase serves as a high-affinity receptor for the Cry48Aa toxin of *Lysinibacillus sphaericus* [53]. Similarly, in *Anopheles gambiae*, an alpha-glucosidase located on the midgut brush border membrane functions as a high-affinity receptor for the Cry48Aa toxin of *L. sphaericus* [50]. Hence, alpha-glucosidases may be cell surface receptors for certain insect viruses. Protein–receptor interactions are often intricate and frequently depend on adaptor proteins or ligands to assemble stable complexes that enhance binding affinity and facilitate downstream signaling [54]. The 14-3-3 protein family functions as signaling adaptors in downstream pathways and trafficking [55] and engages with various receptors [56]. The 14-3-3 epsilon protein we identified in this study may similarly act as an adaptor to improve binding efficiency and mediate downstream signaling; however, this hypothesis warrants further investigation.

Considering our findings, we hypothesize that when DnCPV-23 is ingested orally by the host, polyhedra degrade within the host midgut. The exposed spike protein VP3 recognizes and interacts with alpha-glucosidase on the host cell membrane, presumably with the assistance of adaptor proteins or ligands, thereby facilitating viral attachment and internalization. The identification of MsAGL as an entry factor for DnCPV-23 may offer new perspectives for the development of horticultural plant protection strategies against *D. nerii*. Our study provides a molecular basis for designing intervention measures, such as RNAi-based silencing of MsAGL in pest populations or the development of recombinant viral formulations with enhanced viral binding affinity. Such approaches could potentially improve the efficacy of DnCPV-23 as a biocontrol agent and contribute to integrated pest management in horticultural plant protection.

It is important to acknowledge several limitations of this study. First, our findings primarily rely on the alternative host cell line QB-Ms2-2 from *M. sexta*. While this model supports DnCPV-23 infection, potential differences from the natural host *D. nerii* must be considered. The role of MsAGL could be further verified in non-susceptible cell lines (e.g., human 293T, Vero, or CHO-K1 cells). Second, although MsAGL is a key entry factor, the detailed mechanism of the VP3–MsAGL interaction remains unclear, and potential co-factors or additional participating proteins warrant investigation. Third, the in vivo relevance of our cellular data requires validation through field bioassays though preliminarily supported by larval neutralization assays. Therefore, future studies should therefore focus on: (1) confirming the function of homologous alpha-glucosidases in other cell systems; (2) identifying possible co-receptors or ligands involved in VP3–MsAGL binding; and (3) conducting field trials to evaluate the feasibility and environmental safety of MsAGL-targeting strategies. These efforts will provide a more comprehensive understanding of DnCPV-23 infection.

## 5. Conclusions

This study revealed that the structural protein VP3 is responsible for DnCPV-23 attachment to host cells, with the interaction between VP3 and MsAGL mediating viral entry into host cells. Considering that MsAGL also neutralizes viral infections of host larvae, we propose that for *M. sexta*, an alpha-glucosidase functions as an entry factor for DnCPV-23. These results may form the basis of future research on the mechanisms through which CPVs enter susceptible hosts.

## Figures and Tables

**Table 1:** Primers used in this study.

Primer Name	Primer Sequence (5′ to 3′) *	Target Gene
GAPDH-F	CAAAGTCATTTCCAACGCCT	*GAPDH*
GAPDH-R	CGGTTTTCTGTGTAGCAGTGG	
qPH-F	GTCCGCCAATACTCTCAG	*S10*
qPH-R	CGTAGTCCATCGTCAATCA	
DnVP2-F	TCGAGCGGCCGCATCGTGACATGAGTGAGATAACTATATATGCCCGA	*S3*
DnVP2-R	TAGTACTTCTCGACAAGCTTTTAACTAGCACGCCGAACGTA	*S3*
DnVP3-F	TCGAGCGGCCGCATCGTGACATGTTTATAGAGATCCAATCAA	*S4*
DnVP3-R	TAGTACTTCTCGACAAGCTTTTAGTATACCATCCATCCTCTATTATCATG	*S4*
DnVP4-F	TCGAGCGGCCGCATCGTGACATGATGCTCTACGCCATTGAC	*S5*
DnVP4-R	TAGTACTTCTCGACAAGCTTTTAATCACGCATTAAGCCAATGACA	*S5*
DnVP5-F	TCGAGCGGCCGCATCGTGACATGCTTTTCACAATAAACATTCGTGC	*S7*
DnVP5-R	TAGTACTTCTCGACAAGCTTTTAGTTACTCCTACTCCGCATCACTG	*S7*
DnVP6-F	TCGAGCGGCCGCATCGTGACATGGAAGAAACCCGATCAGTCA	*S8*
DnVP6-R	TAGTACTTCTCGACAAGCTTTTAGTCAATGATAACGCTTCACCAGTGCAT	*S8*
MsAGL-F	GACACCACCACCACCACCACATGTCGGAGCCGCGGAAAAAC	*MsAGL*
MsAGL-R	CCGTCATGGTCTTTGTAGTCCTGCTCCGCGCTTGTCTTGG	*MsAGL*
MsAGL-EXR-F	TCGAGCGGCCGCATCGTGACGAGGAGCTGATGAAGTATGCGGATGA	*MsAGL-EXR*
MsAGL-EXR-R	TAGTACTTCTCGACAAGCTTTTAGGCTCCGCGCTTGTCTTGG	*MsAGL-EXR*
siMsAGL sense	CGAGCUUGAUGCUAACAAACG	*MsAGL*
siMsAGL anti-sense	UUUGUUAGCAUCAAGCUCGAU	
siGFP sense	UUCUCCGAACGUGUCACGUTT	*GFP*
siGFP anti-sense	ACGUGACACGUUCGGAGAATT	

*: The homologous arm sequences were underlined.

**Table 2:** Candidate HBBMV proteins that interact with VP3.

UniProt Accession	Description	Molecular Mass (kDa)	Score *	Sequence Coverage (%) **
A0A922CEJ9	alpha-glucosidase	67.332	452	36.8
A0A921ZED7	Mannosyl-oligosaccharide glucosidase	91.306	166	16.8
D1LYK0	60S acidic ribosomal protein	34.198	244	31.2
A0A921YKX4	ATP-dependent RNA helicase	69.743	228	20.5
A0A9R0EGJ2	Membrane-associated progesterone receptor	19.16	69	9.4
A0A921YYQ7	Calcium-transporting ATPase	107.929	197	21.1
A0A921ZED7	Mannosyl-oligosaccharide glucosidase	91.306	161	22.7
A0A921YUM2	Eukaryotic translation initiation factor 3 subunit D	69.252	323	31.2
A0A922CIN7	Elongation factor 1-alpha	50.423	316	19.7
A0A921Z1J7	Voltage-dependent L-type calcium channel subunit alpha	210.480	52	6.7
Q9U5N0	V-type proton ATPase subunit H	55.024	281	20.3
A0A921YZW4	14-3-3 epsilon protein	26.99	248	30.8
A0A921ZM62	Sorting nexin	63.666	104	9.6

* Similarity between the peptide obtained by LC-MS/MS and the peptide in the UniProt database according to the Paragon algorithm. A higher score reflects identification by multiple high-quality unique peptides and broad sequence coverage, thus providing robust support for the protein’s presence as determined by LC-MS/MS. ** Amino acid sequence coverage for identified proteins.

**Table 3:** First assay of the neutralizing effect of MsAGL on the DnCPV-23 infection of *D. nerii*.

Group	Mean Survival Time (d)	χ^2^	*P*
CK	13.125 (11.951, 14.299)	41.592	<0.001
MsAGL	13.042 (11.968, 14.116)	44.440	<0.001
DnCPV-23	3.792 (3.480, 4.103)	-	-
BSA + DnCPV-23	9.500 (7.645, 11.355)	0.246	0.620
MsAGL + DnCPV-23	7.552 (6.575, 8.529)	26.831	<0.001

**Table 4:** Second assay of the neutralizing effect of MsAGL on the DnCPV-23 infection of *D. nerii*.

Group	Mean Survival Time (d)	χ^2^	*P*
CK	13.708 (13.149, 14.268)	46.611	<0.001
MsAGL	13.792 (13.392, 14.191)	46.611	<0.001
DnCPV-23	3.333 (3.007, 3.660)	-	-
BSA + DnCPV-23	3.500 (3.211, 3.789)	0.625	0.429
MsAGL + DnCPV-23	9.785 (8.033, 11.717)	30.302	<0.001

**Table 5:** Comparison of animo acid sequences of MsAGL and corresponding animo acid sequences in other species.

Species Name	Amino Acid Similarity (Identity)
*S. litura*	70.96% (55.14%)
*Trichoplusia ni*	75.41% (60.10%)
*S. frugiperda*	71.13% (55.79%)
*S. exigua*	66.23% (50.24%)
*B. mori*	60.59% (46.74%)
*Helicoverpa armigera*	62.87% (46.58%)
*Galleria mellonella*	70.55% (55.18%)

## Data Availability

The original contributions presented in this study are included in the article/Supplementary Materials.

## Supplementary Materials

The following supporting information can be downloaded at: https://www.mdpi.com/article/10.3390/v18030293/s1, Figure S1: Recombinant protein expression in this study.
