# Supplementary material for: Alpha-Glucosidase of Manduca sexta Is an Entry Factor for Daphnis nerii Cypovirus-23
Source: Viruses. 2026 Feb 28;18(3):293. doi: 10.3390/v18030293 (PMC13030620; doi:10.3390/v18030293)
Supplement: Supplementary file 1 [file viruses-18-00293-s001.zip › Supplementary materials revised.pdf]

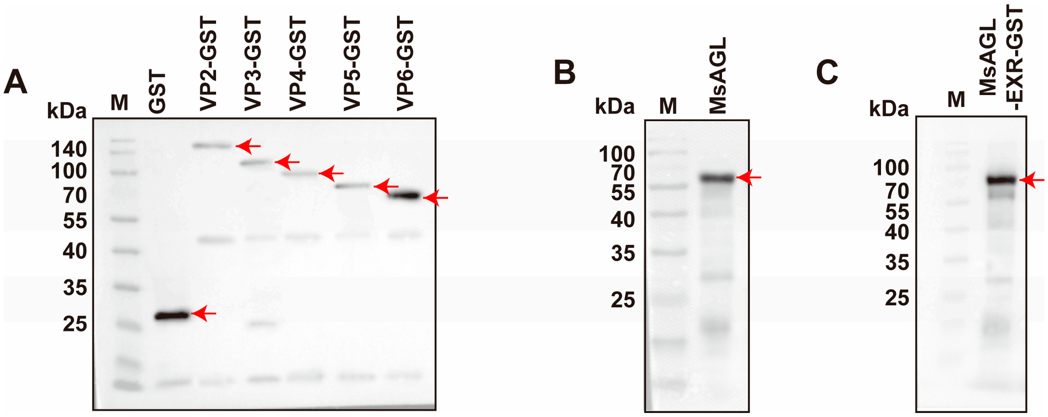


**Figure S1.** Recombinant protein expression in this study. (**A**) GST and GST-tagged viral structural proteins expressed in Sf9 cells; (**B**) Expression of MsAGL in Sf9 cells; (**C**) Expression of GST-tagged MsAGL-EXR in Sf9 cells. Red arrows indicate the expected bands corresponding to the target recombinant proteins.
